# Delineating three-dimensional behavior of uveal melanoma cells under anchorage independent or dependent conditions

**DOI:** 10.1186/s12935-024-03350-0

**Published:** 2024-05-23

**Authors:** Alicia A. Goyeneche, Jade M. E. Lasiste, Mohamed Abdouh, Prisca Bustamante, Julia V. Burnier, Miguel N. Burnier

**Affiliations:** 1 The MUHC-McGill University Ocular Pathology & Translational Research Laboratory, Research Institute of the McGill University Health Centre, Montreal, Canada; 2 Cancer Research Program, Research Institute of the McGill University Health Centre, Montreal, Canada; 3 Experimental Pathology Unit, Department of Pathology, McGill University, Montreal, Canada; 4 Department of Oncology, McGill University, Montreal, Canada

**Keywords:** Uveal melanoma, Three-dimensional in vitro culture methods, Anchorage-dependent, Anchorage-free, Multicellular tumor structures, VEGF, Matrigel

## Abstract

**Background:**

Although rare, uveal melanoma (UM) is a life-threatening malignancy. Understanding its biology is necessary to improve disease outcome. Three-dimensional (3D) in vitro culture methods have emerged as tools that incorporate physical and spatial cues that better mimic tumor biology and in turn deliver more predictive preclinical data. Herein, we comprehensively characterize UM cells under different 3D culture settings as a suitable model to study tumor cell behavior and therapeutic intervention.

**Methods:**

Six UM cell lines were tested in two-dimensional (2D) and 3D-culture conditions. For 3D cultures, we used anchorage-dependent (AD) methods where cells were embedded or seeded on top of basement membrane extracts and anchorage-free (AF) methods where cells were seeded on agarose pre-coated plates, ultra-low attachment plates, and on hanging drops, with or without methylcellulose. Cultures were analyzed for multicellular tumor structures (MCTs) development by phase contrast and confocal imaging, and cell wellbeing was assessed based on viability, membrane integrity, vitality, apoptotic features, and DNA synthesis. Vascular endothelial growth factor (VEGF) production was evaluated under hypoxic conditions for cell function analysis.

**Results:**

UM cells cultured following anchorage-free methods developed MCTs shaped as spheres. Regardless of their sizes and degree of compaction, these spheres displayed an outer ring of viable and proliferating cells, and a core with less proliferating and apoptotic cells. In contrast, UM cells maintained under anchorage-dependent conditions established several morphological adaptations. Some remained isolated and rounded, formed multi-size irregular aggregates, or adopted a 2D-like flat appearance. These cells invariably conserved their metabolic activity and conserved melanocytic markers (i.e., expression of Melan A/Mart-1 and HMB45). Notably, under hypoxia, cells maintained under 3D conditions secrete more VEGF compared to cells cultured under 2D conditions.

**Conclusions:**

Under an anchorage-free environment, UM cells form sphere-like MCTs that acquire attributes reminiscent of abnormal vascularized solid tumors*.* UM cells behavior in anchorage-dependent manner exposed diverse cells populations in response to cues from an enriched extracellular matrix proteins (ECM) environment, highlighting the plasticity of UM cells. This study provides a 3D cell culture platform that is more predictive of the biology of UM. The integration of such platforms to explore mechanisms of ECM-mediated tumor resistance, metastatic abilities, and to test novel therapeutics (i.e., anti-angiogenics and immunomodulators) would benefit UM care.

**Supplementary Information:**

The online version contains supplementary material available at 10.1186/s12935-024-03350-0.

## Background

Uveal melanoma (UM) is a rare disease but is the most common primary ocular malignancy in adults. UM arises through malignant transformation of uveal melanocytes that lay throughout the uveal tract (i.e., a highly vascularized structure with loose connective tissue comprising the iris, the ciliary body, and the choroid. Approximately 4% of UM presentations are established in the ciliary body, 90% of UM are choroidal and only 6% are confined to the iris [1, 2].

Ocular treatment promotes preservation of the eye and vision, and comprises radiotherapy, laser therapy, and surgical resection. Enucleation of the eye is reserved to cases where the tumor covers more than half of the globe and cases with large extraocular extension [3]. Despite satisfactory control of the ocular disease, more than half of patients develop fatal metastasis with an overall survival rate less than 1 year [1–4]. An autopsy population-based study found the liver as a site of metastatic disease in 93% of the cases of UM, while the most common extrahepatic metastasis sites are the lungs (30%), bone (23%), and skin (17%) [1, 4, 5]. Long-term survival is rare, except for patients with isolated liver metastasis that are amenable to surgical resection. Metastatic disease responds poorly to chemotherapy or targeted therapy, so there are no current treatments available to cure patients at metastatic stages [1–3, 6].

Because of the scarcity of ocular tumor tissue to study UM, preclinical in vivo and in vitro studies on biological aspects of cancer and therapeutic interventions are required to improve disease outcomes [7]. Since the early twentieth century, two-dimensional (2D) culture models, where cells grow in a monolayer adhered on plastic or other adherent surfaces, were the most employed pre-clinical in vitro practices for drug screening or disease modelling. However, this modality of growth does not properly model tumor tissue architecture, mechanical and biochemical signals, as well as cell–cell and cell-extracellular matrix (ECM) communications [8–10]. Indeed, tumors are complex multi-cellular entities with heterogenous cell populations, molecular profiles, and genomic markers. Many of these differences are associated with microenvironmental conditions. For example, cells in small tumors, before the establishment of a vascular network, or in microregions of larger tumors with abnormal vascularization, are exposed to a gradient of oxygen, nutrients, as well as to other physical and chemical stresses. Such microenvironmental changes may explain the heterogeneity of cancer cell populations, different metastatic propensities, and drug resistance [11, 12].

Different in vitro methods are currently available to assemble 3D cell cultures where cancer cells establish cell–cell and cell-ECM interactions, which are categorized in two main classes: anchorage-dependent (AD)- and anchorage-free (AF)-based 3D cell cultures [13]. In AD cultures, cancer cells are seeded or embedded in a 3D platform of a non-cellular component, composed of structural proteins that mimics the ECM [10, 12, 14, 15]. On the other hand, the AF-based 3D-culture known as the multicellular tumor spheroid (MCTS) model was developed in the late twentieth century [12]. The main principle is the formation of MCTS from single-cell suspensions of cancer cells in conventional fetal bovine serum (FBS)–supplemented medium preventing adherence to a substrate and without the supply of exogenous ECM [14, 16]. It represents an attractive 3D platform because it recreates tumor cell heterogeneity features observed in abnormal vascularized tumours in vivo [11, 12].

Hence, the aim of this study was to comprehensively evaluate and assess the biological behavior of well characterized UM cell lines to adapt onto different 3D-culture systems under anchorage-free or anchorage-dependent conditions [17–19].

## Materials and methods

### Uveal melanoma (UM) cells

Six well-characterized UM cell lines with known *GNAQ* or *GNA11* mutations, different population doubling times, and originating from primary, metastatic, or from PDXs were used in this study [7]. MP41 and MP46 were purchased from the American Type Culture Collection (ATCC) and were developed from PDXs of a primary tumor [17–19]. UM 92.1 cells were gifted by Dr. Martine Jager (Leiden University, Netherlands); the cell line was developed from a large extraocular outgrow tumor on the right orbit [18]. MEL270 and OMM2.5 UM cell lines were gifted by Dr. Vanessa Morales (University of Tennessee, USA) and were developed from the same patient. MEL270 cells were derived from a recurrent primary tumor that had been irradiated, whereas OMM2.5 were derived after irradiation and subsequent recurrent metastasis. Finally, MEL285 UM was developed from a large ciliochoroidal melanoma [18, 20]. The identity of the six UM cell lines was certified by short tandem repeats (STR), with five confirmed to bear known UM-driver mutations in *GNAQ* or *GNA11*, and one being wildtype for both genes using droplet digital polymerase chain reaction (ddPCR) (see below) (Additional File 1: Fig.S1). All cell lines were tested free of mycoplasma contamination before use in the study. The experimental data was evaluated within less than ten cell-passaging, and the specific stock passages range between P4 and P31.

### Cell culture conditions

Cells were cultured in UM medium consistent of RPMI-1640 supplemented with 10% fetal bovine serum (FBS), 10 mM HEPES, 2 mM dipeptide form of l-Glutamine (glutagro), 1 mM sodium pyruvate, 100 U/mL penicillin and 100 μg/mL streptomycin (all from Corning, Corning, NY, USA), and 10 μg/mL insulin (SAFC Biosciences, Lenexa, KS, USA). Cells were incubated at 37 ºC under 5% CO_2_ in a humidified incubator. The number of cells to be plated for each experimental approach were assessed by using a TC20 Automated cell counter (Bio-Rad, Irvine, CA, USA).

### Assessment of specific hotspot mutations that characterize each UM cell line

Genomic DNA (gDNA) was isolated from cultured UM cells using the QIAamp DNA Mini Kit (QIAGEN, Germantown, MD, USA) according to the manufacturer’s instructions. The total DNA collected was kept in 200 μl AVE buffer (RNase-free water supplemented with 0.04% sodium azide; Qiagen) and quantified using the Qubit dsDNA HS assay kit on a Qubit 2.0 fluorometer (Thermo Fisher Scientific, Waltham, MA, USA). Droplet digital polymerase chain reaction (ddPCR) was used to assess gDNA through the presence of specific hotspot mutations in the respective cancer cell lines used. Samples were run in triplicates in a C100 thermal cycler (Bio-Rad Laboratories, Hercules, CA, USA) following a protocol reported previously [21]. PCR-grade water (Invitrogen, Waltham, MA, USA) was added to each assay as no template control (NTC) and gBlocks (artificial DNA fragments) were used as positive control. Data were analyzed in Quanta Soft v 1.7.4.

### Anchorage-free methods

#### Hanging drop

Different number of cells (5, 1, 5, 10, and 20 × 10^3^) contained in 20 µl of completed medium were seeded as a drop in the inner side of a 100-mm dish lid. The lid was turned upside down and placed on top of a plate filled with 10 ml of phosphate-buffer saline (PBS). A modified hanging drop method with methylcellulose used 20% of the final volume with 1.2% methylcellulose (Millipore-SIGMA M7027, Burlington, MA, USA). MCTs formation and compaction were documented by phase contrast images using an EVOS XL microscope (Thermo Fisher Scientific) upon 48, 72, or 96 h of incubation at 37 ºC under 5% CO_2_ in a humidified atmosphere.

#### Ultra-low attachment plates (ULAP)

Either 5 × 10^3^ cells contained in 200 µl of completed medium were added to each 96 round-bottom well plates (Corning, #7007), or cells were combined with 20% of the final volume of 1.2% methylcellulose. On day four or day seven, the MCTs that developed in each well were scanned using a 4 × objective in a IncuCyte^®^ S3 Live-Cell Analysis System (Sartorius, Oakville, ON, Canada). The total area of each sphere in µm^2^/image, with or without methylcellulose, was averaged and graphed in quintuplicate.

#### Agarose pre-coated plates

Agarose was dissolved in PBS at 1% w/v and autoclaved for 20 min at 120 °C. Fifty µl of agarose were added to each 96-well plate and allowed to solidify for around 20 min at room temperature (RT) under sterile conditions. In a final volume of 100 µl of medium the appropriate number of cells and a 20% of the final volume of 1.2% methylcellulose were combined.

### Handling and colorimetric staining of multicellular tumor structures

The MCTs developed in ULAP at day 4 were fixed in 4% paraformaldehyde (PFA; Sigma, Burlington, MA, USA) for 20 min at RT. Immobilization of MCTs was facilitated by Histogel™ (Richard-Allan Scientific, Kalamazoo, MI, USA) before paraffin embedding, and followed by 5 µm sectioning before hematoxylin–eosin (H&E) or trichrome-mason staining.

### Imaging assessment of multicellular tumor spheres parameters

The images of MCTs with spherical shape developed on anchorage free methods (hanging drop and agarose coated plates ± methylcellulose) were processed with ImageJ software [22] to estimate area and perimeter. For calculation purposes, the MCTs were considered as circles.

### DNA synthesis of MCTs on ultra-low attachment plates

Assessment of proliferation was evaluated by the incorporation of a thymidine analogue EdU (5-ethynyl-2′-deoxyuridine) which is efficiently incorporated into DNA during active DNA synthesis, and is fluorescently labelled with a bright, photostable Alexa Fluor™ 647 dye (Click-iT™, Thermo Fisher Scientific, C10340). UM cells developed spheres in ULAP in a final volume of 200 µl of medium. On day 3, 100 µl of medium was carefully removed without disturbing the MCTs, and 100 µl of a 2X EdU solution (20 µM) was added. MCTs were incubated at 37 °C for an additional 24 h. On day 4, media was carefully removed, and 100 µl of 4% PFA was added to fix the MCTs for 40 min at RT. Then the Click-it™ reaction was performed as per manufacturer instructions. At the end of the protocol, the nuclei were stained with blue, fluorescent Hoechst 33,342 dye (5 µg/ml) for 30 min at room temperature and protected from light. The Hoechst 33342 solution was removed and the MCTs were washed twice with 1 mL of PBS. The three-dimensional structures were imaged with a laser scanning confocal microscope (LSM780. Carl-Zeiss, Oberkochen, BW, DE), using a Zeiss EC Plan-Neofluor10x/0.3na objective; the red nuclei images were developed with a 633 nm laser, whereas all blue nuclei images were developed with a 405 nm laser.

### Anchorage-dependent methods

Cells were grown using the traditional 2D system on cell culture treated surfaces or in a 3D system as follows: (1) cells on-top of basement membrane extract (BME); or (2) cells embedded within BME. The BME product used was the phenol red free Matrigel matrix (Corning 356237), which is a solubilized basement membrane preparation extracted from the Engelbreth-Holm-Swarm (EHS) mouse sarcoma.

#### UM cells on-top of a layer of basement membrane extracts

Eight well chamber slides were pre-chilled; thereafter, 90 µl of Matrigel were added and kept at 4 °C. The chamber slides were incubated for 30 min at 37 °C to allow the Matrigel to form a gel. Cells were trypsinized to a single cell suspension. Then 0.16 × 10^5^ cells contained in 55 µl of complete medium were added atop the gel into each well. Cells were allowed to attach for 30 min at 37 °C, after which the remaining 55 µl of media with 10% of the final volume of Matrigel (11 µl) was added towards the wall of the plate not to disturb the cells on top of the Matrigel. Cultures were maintained for 4 days.

### UM cells embedded within a layer of basal membrane extracts

Pre-chilled 8-well chamber slides were pre-coated with 30 µl of Matrigel and incubated for 5 min at 37 °C to allow the Matrigel to form a gel. Cells were trypsinized to a single cell suspension. Thereafter, 0.75 × 10^5^ cells were mixed with 150 µl of Matrigel and added onto the precoated surface. The samples were incubated for 30 min at 37 °C to form a gel; then, 20 µl of medium was added to each well. The culture was maintained for 13 days with medium changes every two days.

### Immunofluorescence of UM cells arranged on 2D, and 3D embedded or on-top of a layer of basal membrane

After 4 days of incubation, either on-top of a layer of basal membrane or on classical 2D, or after 13 days of incubation on basal membrane-embedded systems, medium was gently removed, and cells were washed twice with PBS. Cells were fixed for 10–20 min at RT with 4% PFA. The fixative was removed, the cells were washed twice with PBS, and permeabilized with 0.1% Triton X-100 for 5 min at RT. After two washes with PBS, cells were incubated with 1% bovine serum albumin (BSA; Wisent Bioproducts, St. Bruno, QC, Canada) in PBS for 20 min at RT to reduce background. Thereafter, the cells were washed twice with PBS. Two hundred µl of a 300 nM DAPI solution (Life Technologies, Carlsbad, CA, USA) were added for 10 min. After two more washes the cells were incubated overnight at 4 °C with the primary antibody diluted in 1% BSA-PBS. The next day the cells were washed twice with PBS for 3 min, and the appropriate secondary antibody was added and incubated for 30 min at RT protected from light. Primary antibodies used were Melan-A/MART-1 rabbit monoclonal antibody 1/100 (Abcam, Waltham, MA, USA; ab51061), HMB45 mouse monoclonal antibody 1/50 (Santa Cruz Biotechnology, Santa Cruz, CA, USA; Sc-59035). Secondary antibodies used were Alexa Fluor Plus 594 anti-rabbit (A32754) or Alexa Fluor Plus 488 anti-mouse (A32723) at 1/1000 dilution, or Alexa Fluor™ 488 anti-rabbit (A21206) or Alexa Fluor 594 anti-mouse (A21201) at 1/500 dilution (Thermo Fisher Scientific). Images were taken using the Bio-Tek Cytation3 Multi-mode microplate reader (Agilent, Santa Clara, CA, USA).

### Phalloidin/DAPI staining

A stock solution of Alexa Fluor^®^ 594 Phalloidin (Life Technologies, Carlsbad, CA, USA) was diluted from its 6.6 μM at a 1:40 ratio in PBS containing 1% BSA. The cells were incubated for 20 min at RT with a solution containing 5 µL of stock Phalloidin in 200 µl of PBS. At the end of the incubation the staining was removed, and the cells were washed twice with PBS. Thereafter, 200 µL of 300 nM DAPI (Life Technologies) solution in PBS was added. Cells were incubated with DAPI staining for 10 min, washed twice with PBS, and kept at 4 °C in PBS until imaging. Images were taken using the Bio-Tek Cytation3 Multi-mode microplate reader (Agilent).

### Vitality assessment

Cell functionality on adherent conditions was evaluated on 3D embedded cells by the ability of the cells to reduce water-soluble tetrazolium salt to an orange formazan dye. After the pertinent time of incubation, the medium was removed and 100 μl of medium containing 10 μl of the CCK8 reagent kit (Dojindo Molecular Technologies, Kumamoto, Japan) was added. The cells were incubated at 37 ºC under 5% CO_2_ for 2 h. Subsequently, the colorimetric product was measured at 450 nm using an infinite M200 Pro plate reader (Tecan Trading AG, Switzerland). Cells plus medium without CCK8 reagent were used as blank controls. Absorbances were recorded as a direct assessment of enzymatic cell reducing capacity and as an indirect measure of cell functionality or wellbeing [23, 24].

### Enzymatic activity and cell membrane integrity assessment

To access cell vitality or wellbeing and membrane integrity on the spheres developed in anchorage-free manner in a 96 ULAP the Live/Dead™ Viability/Cytotoxicity Kit (Life Technology, Oregon, USA; L3224) was used. One of the reagents used was calcein-AM, which is a cell permeant fluorochrome that when cleaved by cell esterase activity gives rise to a green fluorescence that is an indirect indicator of cell vitality. The second reagent used was ethidium homodimer-1 (EthD-1), which only penetrates the cells when the plasma membrane is compromised and generates a red fluorescence when bound to DNA thus serving as a direct estimation of cell toxicity. On day 4 or day 7 of culture half of the total volume of medium was carefully removed without disturbing the spheres, and 100 µl of 2X Calcein-AM/EthD-1 in PBS with calcium and magnesium (DPBS; Corning) was added, followed by a 30 min of incubation at RT. All bright green or red mean intensity was assessed by scanning with the IncuCyte® S3 Live-Cell Analysis System using a 4 × objective.

### Caspase 3/7 activation evaluation

The activation of caspase 3/7 was assessed to evaluate if the cytotoxic core of MCTs carry features of apoptosis. The CellEvent™ caspase-3/7 green reagent is intrinsically non-fluorescent, as the DEVD peptide inhibits binding of the dye to DNA. However, upon activation of caspase-3/7 in apoptotic cells, the DEVD peptide is cleaved, and the free dye can bind the DNA, generating a bright green fluorescence. The CellEvent™ (Thermo Fisher Scientific R37111) ready to use reagent (25 µl) was added to each well containing spheres on day 4. After 30 min of incubation at 37 °C, images were taken using the Bio-Tek Cytation3 Multi-mode microplate reader (Agilent).

### VEGF levels measurement under hypoxic conditions

VEGF concentration was measured in the supernatant of cell cultures derived from 2 and 3D methods. Initially equal number of cells 0.1 × 10^5^/well for all methods were allowed to form their tumor clusters for 16 h. After which all 2D or 3D formation were maintained on hypoxic chamber conditions (1% O_2_, 5% CO_2_, and balanced nitrogen) for 24 h. Following 24 h of incubation, supernatants were collected, and VEGF levels were determined according to the manufacturer’s instructions. We used a human VEGF Quantikine ELISA kit (R&D System, Minneapolis, MN, USA).

### Statistical analysis

One-way or two-way ANOVA followed by Tukey or Bonferroni post test statistical analysis were performed using the GraphPad Prism 9 (GraphPad Software, La Jolla, CA, USA). All data represent means ± s.e.m. A p value < 0.05 was considered statistically significant.

## Results

### UM cells developed multicellular tumor structures (MCTs) under anchorage-free (AF) culture conditions

To assess the tendency of UM cells to aggregate and to establish cell–cell arrangement in the absence of ECM components, we selected a panel of primary and metastatic UM cell lines with different genomic and growth characteristics (Additional file 1: Fig. S1). The 2D growth and biological properties of these cells has been previously described and, in our study, we used this condition as control [7, 17–20]. We utilized these cell lines to uncover whether they have a common morphological adaptation and growth performance under various anchorage-free 3D culture conditions (i.e., ultra-low attachment plate (ULAP) method, hanging drop (HD) method, or agarose-coated plate (ACP) method).

**Fig. 1:**
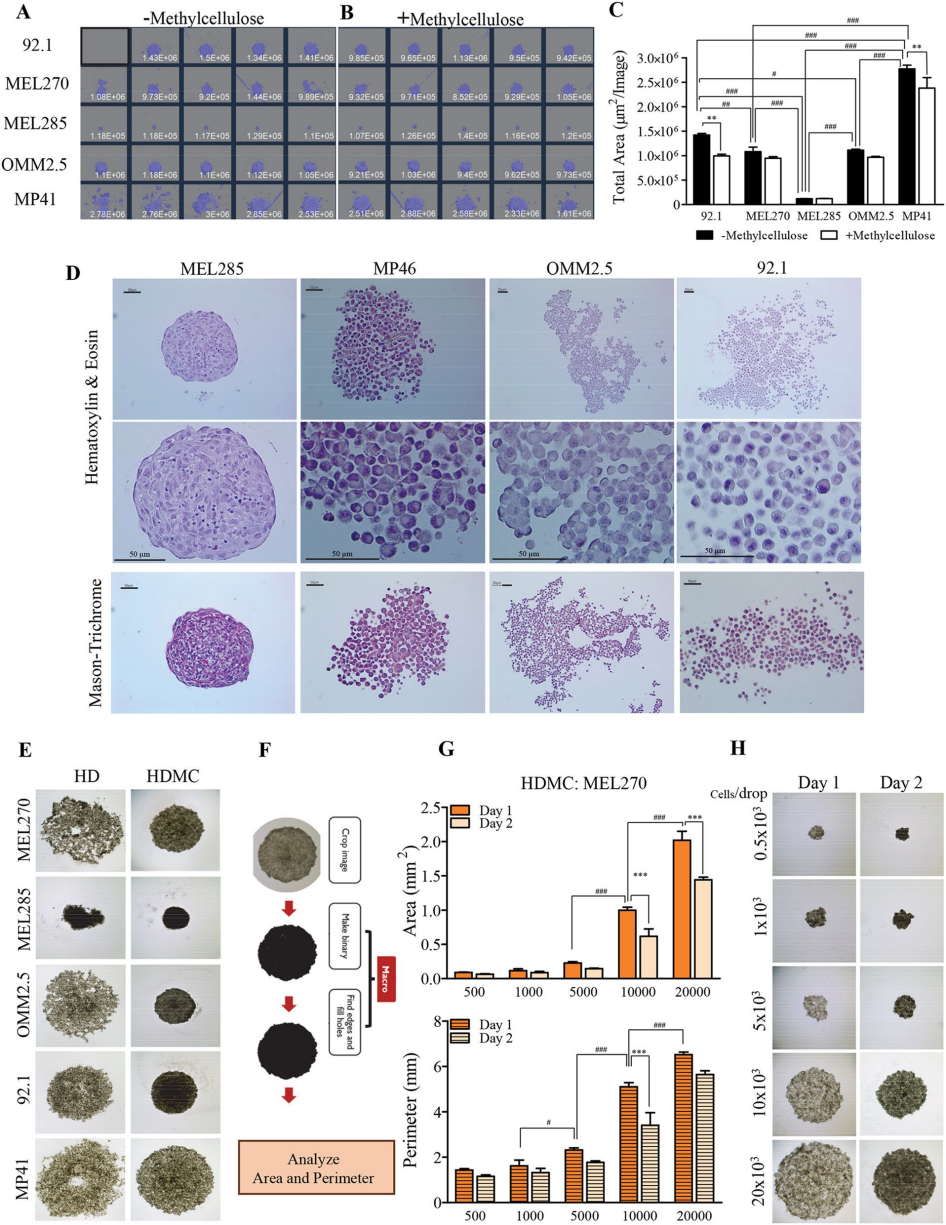
UM cells developed spheroid-like MCTs under AF culture conditions that displayed different behavior. **A**, **B** 5 × 10^3^ UM cells were plated on ULAP, in the presence or absence of MC. Forming MCTs were analysed at day 4 using an IncuCyte System. Figures displayed MCTs scanned at 4 × magnification. **C** Variation of MCTs size as analysed by total area of cultures shown in **A** and **B**. Data are presented as mean ± SEM, (n = 5, ^**^p < 0.01, ^#^P < 0.05, ^##^P < 0.01, ^###^P < 0.001) *for differences in total area between ± MC, and # for differences in total area -MC. **D** Cell–cell interaction and morphological arrangement of MCTs developed at day 4 on ULAP were processed for H&E and Trichrome staining. Representative images showed that UM cells established weak cell–cell interaction, except for MEL285, which developed a tissue like structure. **E** 20 × 10^3^ UM cells were seeded in 20 µl of medium and cultured in HD condition. Representative pictures of the spheres formed on day 4 in the presence (+) or absence (−) of MC are shown. **F**–**H** Formed MCTs were analyzed by their area and perimeter to describe cell density and compactness. **F** The spherical structures formed were considered as particles and analyzed with the macro shown. **G** Graphs of area and perimeter of MEL270 cells that were cultured under HDMC conditions. Note that higher number of cells associated with larger area and perimeter (denoted by #). In contrast, MCTs compaction increased with incubation length (day 2 compared to day 1 denoted by *). Data are presented as mean ± SEM, (n = 5, ^#^p < 0.05, ^###^p < 0.001, ***p < 0.001). **H** Representative MCTs pictures taken on day 1 and 2 with different number of cells

We tested the biological performance of UM cells under ULAP culture condition by seeding 5 × 10^3^ cells per well of a 96 round-bottom well plate and subsequent culture for 4 days. Plated cells started to aggregate and to develop cell–cell arrangements, which further progressed to the establishment of MCTs shaped as spheres of different sizes and degrees of compaction (Fig. 1A). MEL285 cells had the strongest level of compaction as indicated by the reduced area of the spheres, whereas MP41 was the least compact and formed loose MCTs. In between, we found that MEL270 and OMM2.5 were more compact than 92.1 (Fig. 1A, C). Cell–cell interaction and morphological arrangement of MCTs was further assessed by hematoxylin–eosin (H&E) and mason-trichrome staining. Most UM cells established weak cell–cell interaction, failing to conserve the structure during histology processing, except for MEL285, which developed a tissue like structure (Fig. 1D).

To determine the impact of the medium viscosity on the compaction of the formed MCTs, cells were incubated in the presence of methylcellulose (MC). Notably, we observed a significant increase in the compaction of MCTs derived from 92.1 and MP41 cells, but not of those formed from MEL285, MEL270, and OMM2.5 cells (although a slight increase was seen with MEL270, and OMM2.5 cells) (Fig. 1B, C). It is interesting to remark that each cell line seems to follow a cell–cell interaction program of adaptation, reaching its own level of compaction and a particular size that in most cases is independent of the presence of MC (Fig. 1A–D).

In the hanging drop method, UM cells were suspended under gravity in a drop of culture medium (Fig. 1E; left panels). Interestingly, cells developed less compact structures when compared to the ones cultured in the presence of MC (Fig. 1E; right panels). The spheres became more compact with time, and they reach maximal compactness by day 4, more dependably on the presence of MC as observed with both ULAP and HD methods (Fig. 1B, E and Additional file 2: Fig. S2).

To estimate the area and the perimeter of the spherical structures formed in the hanging drops by each cell type, images of individual spheres were considered as particles and analyzed using the ImageJ software (Fig. 1F). The increase in number of cells per drop was associated with a larger area and perimeter, which was significant within the range of drops containing between 5000 cells and 20,000 cells (Fig. 1G, H and Additional file 3: Fig. S3). The tendency of the cells to arrange themselves within a more compact sphere with time is unique for each UM cell-derived MCTs (except for MP41 cells) and is depicted by a decrease in the area and perimeter of the spheres on day 2 versus that formed on day 1 (Fig. 1G, H and Additional file 3: Fig. S3).

Next, 3D cultures with MC on agarose-coated plates were evaluated to determine whether agarose provided a more effective culture milieu than the previously studied ultra-low attachment and hanging drop methods for non-adherent culture conditions. UM cells plated on agarose in the presence of methylcellulose invariably developed spheres like those formed using ULAP and HD methods (Additional file 4: Fig. S4A). Moreover, on day 2, the formed spheres were more compact, and like with the other matrix-free methods, MEL285 cells formed the more compact spheres while MP41 cells were the least compact (Additional file 4: Fig. S4A).

The compaction behavior of MCTs generated using the hanging drop and agarose coated methods was evaluated by area and perimeter (Additional file 4: Fig. S4B and S4C). In general, UM cell-derived spheres using the hanging drop with methylcellulose were more compact than those formed on agarose coated plate (Additional file 4: Fig.S4B–D).

Together, these data show that cultured UM cells maintained the potential to organize as multicellular tumor structures shaped as spheres using different anchorage-free methods. Regardless of the methods utilized, we conclude that intrinsic characteristics of the UM cells define the level of aggregation and compaction they could achieve when they are challenged to live in such environments.

### UM cell spheres developed on anchorage-free environment maintained their vitality and proliferative potential

**Fig. 2:**
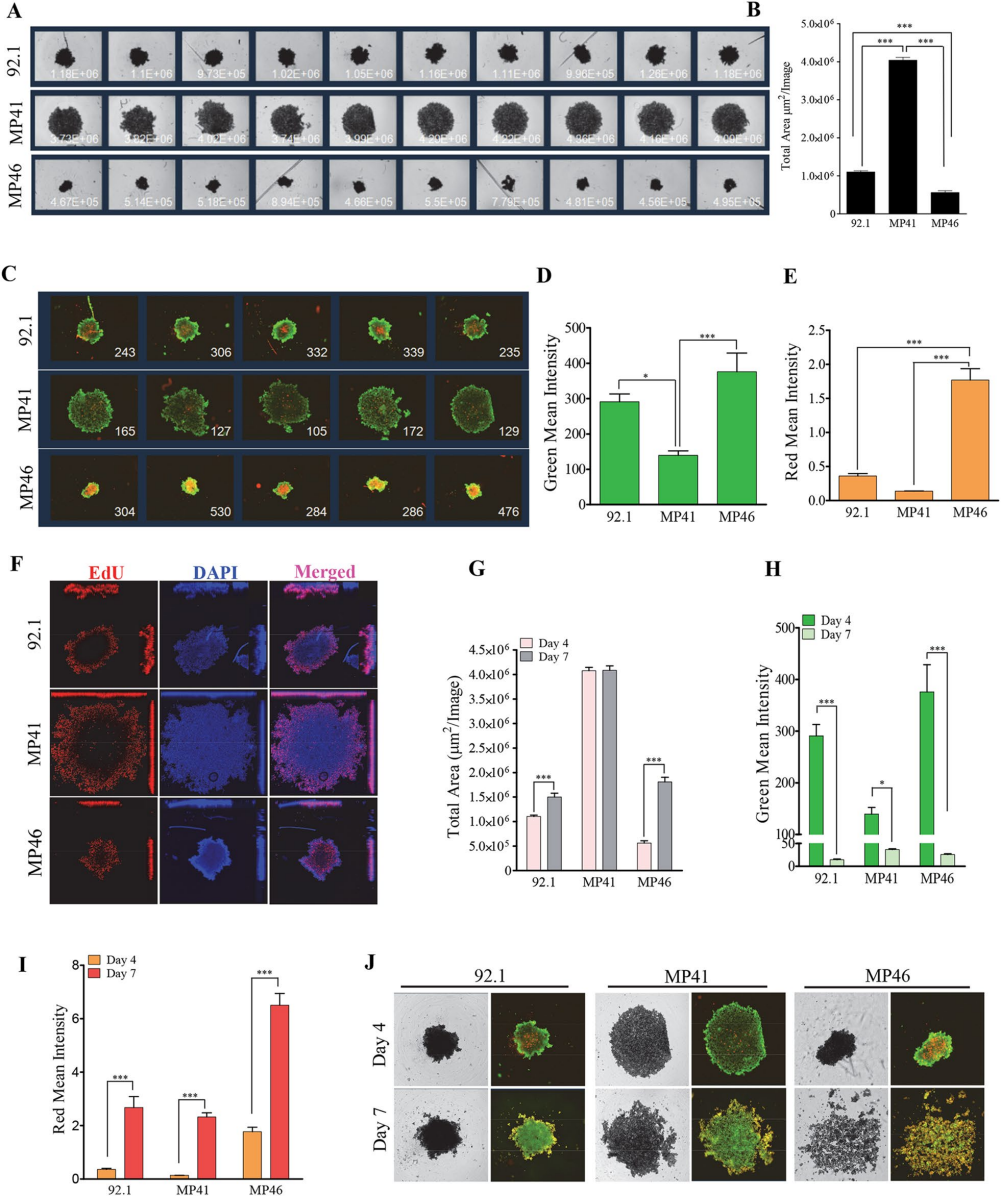
UM cell forming MCTs developed on AF environment maintained their vitality and proliferative potential. **A** UM cells were maintained on ULAP method. Scans using an IncuCyte System at day 4 are shown (4 × magnification). **B** Size of formed MCTs (shown in **A**) as displayed by total area. Data are presented as mean ± SEM, (n = 10, ***p < 0.001). **C** Representative image showing the vitality of formed MCTs as measured by the Live/Dead probes. **D**, **E** Mean fluorescence intensity was measured using an IncuCyte System, where the green mean intensity denotes the esterase enzymatic activity of cells (**D**), while the altered membrane integrity is reflected by the red mean intensity (**E**). Data are presented as mean ± SEM, (n = 5, *p < 0.05; ***p < 0.001). **F** Growing UM cells derived MCTs were analyzed for cell proliferation as measured by the levels of DNA incorporation of the thymidine analogue EdU. Nuclei were counterstained with Hoechst 33342 (blue). Note the active DNA synthesis occurring at the periphery of the MCTs. (G-I) UM cells-formed MCTs were analysed for their growth (size), and vitality at different time-points (4 days vs. 7 days). Data are presented as mean ± SEM, (n = 5, *p < 0.05; ***p < 0.001). **J** Representative images of formed MCTs using bright field imaging (left panels) or fluorescence imaging displaying the overlap of green (vitality probe) and red (membrane damage probe) channels (right panels)

Using the ULAP method, we evaluated the behavior of 92.1, MP41 and MP46 cells and found that MP46 cells formed MCTs on day 4 that were more compact than those formed by 92.1 and MP41 cells (Fig. 2A, B). In these structures, UM cells maintained their vitality in a graded manner with those in the periphery having retained their active enzymatic activity, while those in the core experiencing more cell damage. Notably, 92.1 and MP46 cells displayed a better vitality compared to MP41 cells, but MP46 cells acquired higher membrane integrity damage in the center (Fig. 2C–E). We next compared vitality between UM cells forming MCTs cultured without and with methylcellulose. Cell vitality was similar in both conditions and invariably of the cell line used except for MEL285 cells that formed the more compacted spheres with a highly active cells in the periphery and more damaged cells in the sphere core (Additional file 5: Fig. S5).

In parallel, we assessed the MCTs cultures for cell proliferation potential. Interestingly, and like our observations regarding cell vitality proliferation is mainly taking place at the edges of the analysed 92.1, MP41, and MP46 UM cell-derived spheres (Fig. 2F).

In contrast, nuclei in the core of the MCTs are less actively synthesizing DNA and there is less nuclear density specially in the more compact spheroids formed from MP46 cells (Fig. 2F). In line with this observation, we found that the core of the formed spheroids exhibited cells with apoptotic features (i.e., Caspase 3/7 activation), with cells forming the more compacted MCTs having the highest levels of apoptosis (i.e., MEL285 and 92.1 cells) (Additional file 6: Fig. S6A).

To get more insights on the long-term behaviors of UM cell derived MCTs, we assessed total area, cell vitality, and membrane integrity in the multicellular structures formed from 92.1, MP41, and MP46 cells. We established cultures for 7 days and we compared the behaviors of generated MCTs between day 4 and day 7 timepoints. The more consistent finding was a decrease in membrane integrity on day 7 as compared to day 4, and this was independent of the presence or absence of MC (Fig. 2I; Additional file 6: Fig. S6D and S6H). Total area of the sphere, as a measure of cells growth, did not change between days 4 and 7 of culture, except for the MCTs derived from 92.1 and MP46 cells that show an apparent increase in total area on day 7 (Fig. 2G). The increase in the total area was found in the spheres derived from 92.1, MEL270, and MP41 cells, in the presence of MC but not in the absence of MC (Additional file 6: Fig. S6E and S6I). A significant increase in cell toxicity on day 7 was independent of total area chances and regardless of MC (Additional file 6: Fig. S6D and S6H). We conclude that when extending the time of incubation from 4 to 7 days, the area of the MCTs is ill-defined as some structures become less compact while displaying less vitality and more cytotoxicity (Fig. 2H–J). In general, cell vitality was maintained during the seven days regardless of the absence or presence of MC, except for MCTs derived from OMM2.5 in the presence of MC that reached higher vitality on day 7 but accompanied with also higher toxicity (Additional file 6: Fig. S6C, S6G and S6H). While cell toxicity increases on day 7, regardless of the presence of methylcellulose in spheres formed from 92.1, OMM2.5, and MP41 cells. The increase in cell toxicity for MEL270 spheres was only significant in the presence of MC. Interestingly, MCTs formed from MEL285 cells on day 7 maintained the level of toxicity reached on day 4, regardless of the presence or absence of MC (Additional file 6: Fig. compare S6D to S6H).

Together, these data show that UM cell formed multicellular tumor structures that under anchorage independent conditions reached a maximum size on day 4 of cell culture that does not increase with time, besides an active DNA synthesis and maintenance on cell vitality during the 7 days for most analyzed cells. However, during that time, cell toxicity increases, suggesting that the spheres reached an equilibrium between cell growth and cell toxicity. Cell death happened in a graded manner that progressed from the core to the periphery of the formed MCTs during the 7 days of culture, most probably due to a decrease oxygen and nutrients availability. We conclude that 4 days culture is optimal for the study of UM cell derived MCTs.

### Dissimilar morphological adaptation with a comparable level of vitality on anchorage dependent (AD) conditions

All six UM cell lines were challenged to adapt, adhere, and arrange to the classical 2D tissue culture treated surfaces, or to anchorage-dependent3D conditions where cells are either embedded or seeded on top of a layer of basement membrane extract Matrigel (BME).

**Fig. 3:**
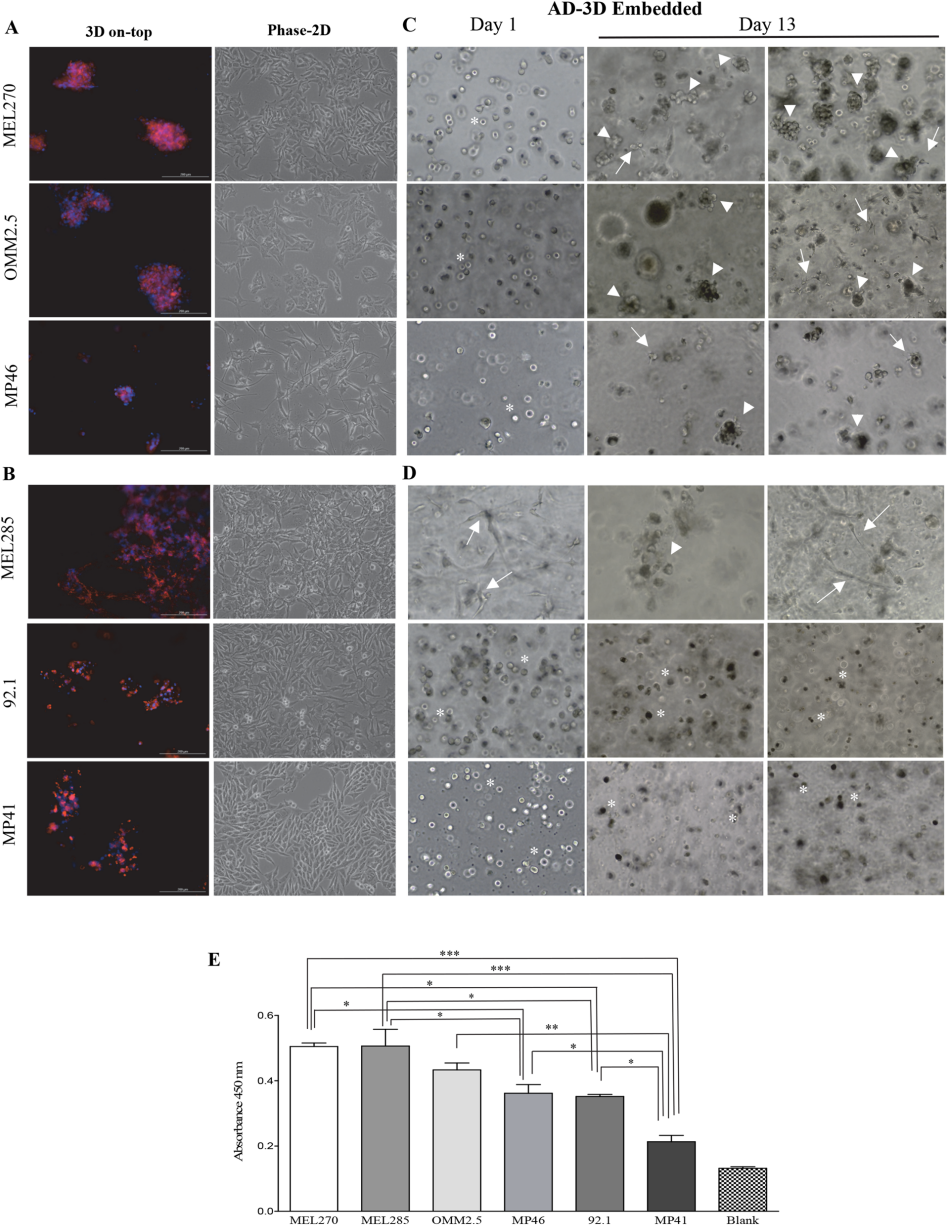
Dissimilar morphological adaptation with a comparable level of vitality on AD conditions. **A**, **B** Arrangement of UM cells seeded on top of BME (left panels) compared to cells cultured on standard 2D setting (right panels). **A** For UM cells group 1 (MEL270, OMM2.5 and MP46), note that cells cultures on top of a BME formed irregular tight aggregates of different sizes as observed by rounded filamentous-actin distribution and nuclear disposition in the space. In contrast, the same cells maintained in standard 2D conditions acquired stretched and flat appearance as observed on representative phase contrast images. **B** For UM cells Group 2 (MEL285, 92.1, and MP41), note that cells adhere on top of BME forming flat patches with more affinity to the substrate than to cell–cell interaction shown by phalloidin/DAPI staining in 3D (left panels). Right panels: similar morphological arrangements onto 2D treated surfaces shown with phase contrast images. **C**–**E** Arrangement of UM cells embedded within a layer of BME. After 13 days in culture, morphological adaptation (**C**, **D**) and vitality (**E**) was evaluated. **C**, **D** Phase contrast images illustrate how cells arranged from day 1 to day 13. **C** UM cells were rounded at the beginning showed by asterisks. Arrowheads indicating clusters of irregular shapes, and different sizes that MEL270, OMM2.5, and MP46 cells formed. Arrows depicting few cells from OMM2.5 and MP46 that are capable to stretch acquiring a mesenchymal phenotype. **D** Right at the beginning MEL285 cells stretched and flattened within the BME indicated by arrows, while 92.1 and MP41 remain isolated and rounded indicated by asterisks. **E** UM cell enzymatic activity as measured by the reduction capacity of the CCK8 probe. Note that regardless of cell arrangements, MEL270, OMM2.5 and MEL285 cells displayed comparable vitality. Lower vitality was observed for MP46 and 92.1 cells, and MP41 cells have the lowest reducing enzymatic capacity of all UM tested. Data are presented as mean ± SEM, (n = 3, *p < 0.05; **p < 0.01; ***p < 0.001)

First, we analyzed the behavior of UM cells seeded on top of a layer of BME. Cellular arrangement was evaluated based on actin distribution, nuclei disposition, and by disposition on 2D culture treated surfaces. UM cells were classified in three groups based on arrangements established on 3D and 2D anchorage-dependent conditions. Group 1 is composed of MEL270, OMM2.5, and MP46 cells, which adapt on top of a layer of BME forming irregular tight aggregates of different sizes as observed by rounded filamentous-actin distribution and nuclear disposition in the space (Fig. 3A; left panels). In contrast, the same cells in 2D acquired stretched and flat appearance as observed on the phase contrast images (Fig. 3A; right panels). Group 2 is composed of MEL285, 92.1, and MP41 cells that adhere on top of the layer of the BME forming flat patches with a higher preference of binding to the substrate than to each other so resembling the morphological arrangements onto 2D treated surfaces (Fig. 3B; left vs. right panels). Cell–cell and cell-BME interaction is shown by phalloidin/DAPI staining in 3D or by phase contrast images on 2D conditions (Fig. 3A and3B. Group 3 consists of the flat appearance acquired onto a 2D plastic treated surface and analyzed by phase contrast images (Fig. 3A, B right panels), and by fluorescent images (Additional file 7: Fig. S7B and S7C). Morphological 2D adaptation reveals those resembling a normal choroidal melanocyte phenotype acquiring a dendritic to a fibroblast like appearance represented by 92.1, MEL285, and MP46 cells, and those with an epithelioid phenotype represented by MEL270, OMM2.5 and MP41 cells.

In parallel, we also analyzed UM cells embedded within a layer of BME. In this case, UM cells adapted for 13 days after which they were morphologically and functionally tested. MEL270, OMM2.5, and MP46 cells established cell–cell interactions forming tight clusters of different sizes similarly than those developed over the top of the BME. The morphological arrangements were characterized by the features presented in phase contrast images and by the disposition of actin filaments and nuclei (Fig. 3C; and additional file 7: Fig. S7A). We observed UM cell lines forming irregular clusters, but not spherical structures like in the anchorage independent models, furthermore few cells were found to be able to stretch within the Matrigel acquiring a mesenchymal phenotype (Fig. 3C, and additional file 7: Fig. S7A). On the other hand, MEL285 cells stretched and flattened right away within the layer of BME (Fig. 3D, Day 1 vs. Day 13), like they did in 3D on top of the BME or in 2D cultures (Fig. 3B), but they did not form clusters, suggesting that these cells had more affinity to the BME than to one another. In contrast, 92.1 and MP41 cells did not develop cell–cell interactions and remain isolated and rounded observed in the phase images (Fig. 3D) compare day 1 vs. day 13 scarce cells might try to be stretched in Matrigel. The differences of UM cells adaptation within the Matrigel can be noted by contrasting day 1 and day 13 on phase images and phalloidin staining (Fig. 3C, D and Additional file 7: Fig. S7A).

Despite a very different way of adaptation when embedded within a layer of BME, UM cells maintain their metabolic activities (Fig. 3E). The enzymatic capacity revealed that MEL270, OMM2.5 and MEL285 cells reached comparable vitality. Another example of different cell arrangements, but similar level of vitality was that of MP46 and 92.1 cells. UM-MP41 cells had the lowest reducing enzymatic capacity of all UM tested thus representing the cells with less vitality of all cells analyzed in the present study.

Taken together, these data show that under adherent conditions, UM cells displayed a diverse morphological adaptation: some UM cells adapted similarly either in 3D on top of the BME or embedded within the BME where they form tight clusters in space, while others developed flattened structures on top of the BME, like in 2D, while others were unable to establish cell–cell interactions and were isolated within the BME. Regardless of being in 2D or 3D environments, UM cells in anchorage-dependent manner maintained the expression of melanocytic markers MelanA/Mart-1 and HMB45 (Additional file 7: Fig. S7B-D).

### UM cells cultured under 3D culture environment efficiently released VEGF following hypoxia

**Fig. 4:**
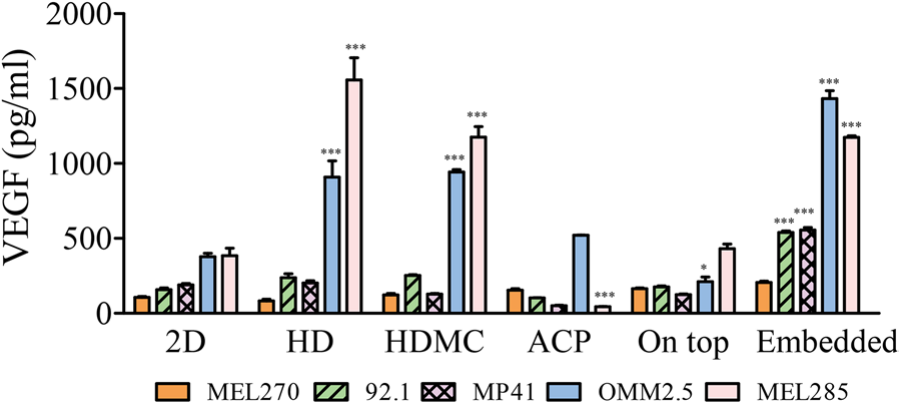
UM cells cultured under 3D culture environment efficiently released VEGF following hypoxia. Initially equal number of cells for all methods were allowed to form their tumor clusters for 16 h. After which all 2D or 3D formation UM cells were maintained in hypoxic atmosphere (1% O_2_) for 24 h. The levels of released VEGF was measured in the supernatant. The differences analysed were against the VEGF level reached on 2D condition. Data are presented as mean ± SEM, (n = 3, *p < 0.05; ***p < 0.001)

To characterize the influence of 2D and 3D culture conditions on the production of the angiogenic factor VEGF, Same number of UM cells were maintained in hypoxic environment (1% O_2_) for 24 h. Irrespective of the culture settings, all UM cells released different levels of VEGF under hypoxic conditions. UM cells maintained under 3D culture environment displayed increased VEGF production compared to those cultured in 2D condition (Fig. 4). Moreover, regardless of cells standing on anchorage-dependent or in anchorage-free conditions, the 3D method environment was sufficient to enhance VEGF release. UM cells adherent on top of a BME and the non-adherent cells on top of agarose coated plate did not induce further release of VEGF than the adherent cells on 2D conditions. The greatest response in terms of VEGF production was observed in cells maintained in 3D on anchorage-dependent conditions (i.e., embedded within the BME) or when cultured in anchorage-free conditions as represented by the hanging drop in the presence or absence of methylcellulose (Fig. 4).

## Discussion

Uveal melanomas are classified into spindle, epithelioid and mixed cell types based on their cellular morphology. A large percentage of spindle cells in the tumor denotes a better survival, whereas the epithelioid phenotype is related to metastasis and a poor prognosis. This cellular heterogeneity might be attributed to the fact that melanocytes originate from a highly plastic cell of the neural crest. Another feature of plasticity associated with risk of metastasis on UM is the presence of vascular loops designated as vascular mimicry. A subset of tumor cells, with stem cell properties, are capable of mimicking vascular channels independently of endothelial cells and pericytes [25].

Tumor complexity in vivo cannot be covered by a single experimental model, and it is a common practice in basic research to use several approaches from cell-culture in vitro assays to animal models for early drug screening, disease modelling, and to understand molecular mechanisms underlying the biology of tumors [26].

Short term studies with fresh primary samples are of relevance as cells can reflect their original characteristics better, but the source of these samples is scarce since local treatment of primary UM has moved towards eye preservation [2]. Development of UM cell lines from primary tumors has a success rate of 3% [17]. Moreover, in vitro cell selection in some cases leads to the outgrowth of UM cell lines that do not completely reflect the genetic alterations found in the primary tumor [7, 17, 18].

In this study, we evaluated different 3D-culture systems, which are known to mimic physical and spatial cues reflecting tumor cytoarchitecture and providing an intermediate pre-clinical platform to advance in vivo tumor performance [27].

We characterized the different morphological arrangements and biological performances of different UM cells upon challenges to adapt onto anchorage-free or anchorage-dependent conditions.

A high risk of metastasis in UM is associated with copy loss on chromosome 3, and deficiency of BAP1 protein expression. Our study contains cells lines with typical *GNAQ, GNA11* mutations, with and without expression of BAP1 protein. These cells originate from a variety of sources such as primary, patient-derived tumor xenograft, and metastatic sites [17, 18]. However, one of the limitations of the study is that most of the UM cells have normal copies of chromosome 3, and short-term culture of normal choroidal melanocytes were not included on the comparison.

We found that UM cells in suspension and prevented from adhering to a substrate regardless of the AF method used, organized themselves as multicellular tumor structures shaped as spheres. Each cell line followed a cell–cell interaction program of adaptation that determines the level of aggregation and compaction. MEL285 cells had the strongest level of compaction followed by MP46, MEL270 and OMM2.5, and 92.1, while the least compact of all were the MP41 cells. UM cells maintained under AF conditions mirrored the multicellular tumor spheroid model, in which cells collide with each other and are held together by cell–cell contacts developing the phenomenon of aggregation [14, 28]. The compaction phase of the spheroid depends on active surface cellular processes, like intercellular adhesion molecules, cytoskeleton integrins, or the affinity to specific receptors [11, 12, 14, 28].

The H&E staining reveals that only MEL285 cells can establish a tissue-like organization. In the same way, Aughton et al., have shown that spheres from fresh primary UM specimens acquired a well-developed tissue-like organization, while others established loose cell–cell interaction. In our study, UM cells MP46, 92.1, and MP41 presented weak cell–cell interactions but different degrees of compaction.

Primary tumors from which MEL285, MEL270, and MP46 cells lines were derived, lacked expression of BAP1 protein [17, 18]. However, the cell lines MEL285 and MEL270 were reported to have BAP1 expression. The primary tumor, from which MEL285 cells were established, were predominantly spindle type with only 20% epithelioid cells [18]. In a 3D environment, MEL285 cells produce the most compact spheres, and has the highest released of VEGF.

The increase in viscosity achieved by incubating the cells in the presence of the neutral scaffold methylcellulose only significantly added extra compaction to MCTs of 92.1 and MP41 cells when cultured using ultra-low attachment condition (i.e., ULAP). Of interest, MCTs developed in the hanging drop are more compact than those formed on agarose coated plate in the presence of MC.

In general, the presence of MC improved the organization of the spheres in anchorage-free conditions. In the case of the ULAP culture condition, the presence of MC provided homogeneity without altering vitality and toxicity. The shape descriptors (i.e., area and perimeter) were consistent predictors of compaction of the spherical structure.

Assessment of cell vitality, membrane integrity, cell death, and capacity of proliferation after 4 days demonstrated that, regardless of size and degree of compaction, all UM-derived MCTs had two distinct zones:a core zone with cells displaying signs of cytotoxicity (i.e., loss of membrane integrity and vitality, increased apoptosis, and decreased proliferation), and a ring-like peripheral zone where cells presented high vitality and growth. Therefore, UM cells in an AF environment acquired differential cellular behavior resembling microregions of an abnormal vascularized solid tumor in vivo [7, 8, 11]. The spherical symmetry creates a potential gradient of oxygen, nutrients, soluble signals, and cellular waste recreating similarities within a tumour [8]. The comparison of area, vitality, and toxicity between days 4 and 7 of MCTs incubation indicated that although there was active DNA synthesis on day 4 and maintenance of cell vitality throughout the seven days, the MCTs did not consistently increase in size. In contrast, cell toxicity increased with time, suggesting that spheress reach an equilibrium between cell growth and death after 4 days of incubation. We propose that before testing any biochemical parameters or drug efficacy on MCTs, each UM cell line needs to effectively reach its own level of compactness, as similarly proposed by Aughton et al., [29], where some UM cell lines needed between 4 to 7 days and others up to 10 days to reach an equilibrium in their development. In the same study, the authors showed that cells from primary tumors in anchorage-free environments tend to develop spheres that vary in size, and level of compactness. We argue that the increase or decrease in total area per image is not sufficient to claim growth or to evaluate drug response within MCTs. A combination of imaging-based assays for assessing changes in cell proliferation, vitality, and cellular metabolism are necessary to improve cellular assessments of different zones of the MCTs.

Cell seeding density governs spheroid size and shape, impacting the experimental outcome [30]. Based on our findings, a minimum cell density of 5 × 10^3^ cells to a maximum of 20 × 10^3^ were effective in the development of spheres that become compacted after 2 days of incubation. Our studies lead to the recommendation for using 5 × 10^3^ cells in ultra-low attachment plates to optimally assess cellular behavior.

Adaptability of UM cells to extracellular matrix proteins or anchorage-dependent conditions like growing on top of a layer of BME was not identical across the cell lines. We found cells that were arranged as tight aggregates of different sizes (e.g., MEL270, OMM2.5 and MP46) while others developed a flattened structure resembling adaptation to 2D conditions (e.g., 92.1, MEL285, and MP41). UM cells that resemble a 2D phenotype had difficulties establishing cell–cell interactions and remained isolated, rounded, or stretched despite being completely embedded within the basal membrane.

UM cells with epithelioid phenotype presented a vasculogenic mimicry phenotype in 3D on top and are considered to have invasive potential. In contrast, UM cells with spindle phenotype showed low invasive potential and did not form vasculogenic mimicry patterns under 3D conditions, but rather grew in tumor cell aggregates on the matrix surface and formed multicellular spheroids inside the matrix [31]. A recent study injected the same cells into the vitreous of nude mice and showed that the spindle phenotype generated both spindle and epithelioid tumors, while the epithelioid solely generated epithelioid tumors [25].

The vitality of cells as aggregates, like the cases of more epithelioid MEL270 and OMM2.5 cells, was comparable to that reached by the spindle MEL285 cells. A similar level of comparison was established for MP46 and 92.1 cells, having comparable vitality but different capacity to arrange themselves embedded inside the layer of BME.

Further studies are needed to understand what this different biomechanical adaptation means at the level of metastatic potential capacity, for the ability to interact with the tumor microenvironment, the competency to launch angiogenesis, immunomodulation, and therapeutic responses.

The ability of tumors to form new vasculature is a hallmark of cancer progression and metastasis. UM arises in a capillary-reach tissue, and indeed, enhanced angiogenesis is associated with a higher rate of metastasis and mortality in UM [32, 33]. The ability of UM cells to metastasize has been correlated to their ability to support angiogenesis [34, 35].

Vasculogenic mimicry, regarded as a sign of cancer cell plasticity, was initially found in UM and to be related to a poor prognosis [33]. Furthermore, 3D cultures have recently been proposed suited for research on angiogenesis [36–41]. Based on the diverse forms of adaptation of UM cells within AF or AD conditions, we evaluated their capacity to secrete the proangiogenic factor VEGF under hypoxic conditions. It is known that VEGF and other angiogenic factors play an important role in modulating the immune system directly by suppressing dendritic cell maturation, inhibiting T-cell receptor response, and recruiting myeloid derived suppressor cells [42]. Interestingly, UM cells under 3D culture conditions produced higher levels of VEGF compared to those maintained in 2D culture conditions. The greatest response in terms of VEGF production was reached by cells arranged in a 3D environment regardless of being embedded within a matrix of proteins or in non-adherent conditions (e.g., the HD with or without MC).

## Conclusions

In summary, our work provides evidence for the ability of UM cells to recreate tumor cell heterogeneity on anchorage-free and anchorage dependent conditions. This fosters the inclusion of such platform as a preclinical tool to deepen the understanding of the biology of UM. Moreover, the combination of such platforms to explore mechanisms involved in ECM-mediated tumor resistance, and the impact of co-culturing UM cells with fibroblasts, endothelial cells, or immune cells to test new UM therapeutics (i.e., anti-angiogenic interventions and immune modulation).

### Supplementary Information

Additional file 1: Figure S1. Characterization of UM cell lines used in the present study. (A) Table displaying phenotypic and genotypic characteristics of the used UM cell lines. Patients (age and sex), and tissues of origin were extracted from the literature. Expression of typical melanocyte markers as well as known *GNAQ*/*GNA11* mutation were tested in our laboratory. (B) Representative two-dimensional plot of ddPCR showing the *GNAQ/GNA11* mutation signature. Droplets positive to mutant *GNAQ/GNA11* are shown in blue (FAM channel), wildtype *GNAQ/GNA11* positive droplets in green (HEX channel), double positive droplets for mutant and wildtype *GNAQ/11* are presented in orange, and negative droplets are represented in blank. Threshold line is presented in pink.

Additional file 2: Figure S2. The hanging drop method without or with methylcellulose. Representative images on day 2, day 3, and day 4 of different UM cell lines showing that 5 × 10^3^ UM cells contained in 20 µl of medium and suspended under gravity as a single hanging drop developed multicellular structures (n = 3 independent cultures). Less compactness was observed when compared to the MCTs formed in the presence of methylcellulose.

Additional file 3 Figure S3. Area and perimeter to describe cell density and compactness. Contrast of multicellular structures formed after 1 or 2 days using different amounts of cells per drop (hanging drop method with methylcellulose). OMM2.5 cells (A-C), 92.1 cells (D-F), MEL285 cells (G-I), and MP41 cells (J-L) were analyzed. Cell arrangement was evaluated by MCTs Area (A, D, G and J) and Perimeter (B, E, H and K) was depicted for each cell line. Data are presented as mean ± SEM, (n = 3, ^***^p < 0.001, ^#^p < 0.05, ^##^p < 0.01, ^###^p < 0.001). (C) Representative images of MCTs formed on day 1 and day 2. Note that with increased cell number seeded per drop, larger areas and perimeters were obtained denoted by #. Compactness with time, that is unique to each UM-MCTs, is demonstrated with a decrease in areas and perimeters at day 2 when compared to day 1 denoted by *.

Additional file 4: Figure S4. Multicellular structures formed in agarose-coated plates in the presence of methylcellulose. Area and Perimeter comparison with HDMC. (A) UM cells plated on agarose in the presence of MC developed MCTs which were more compact on day 2. (B-C) Compaction behavior of MCTs generated using the HD and ACP methods in the presence of MC. The multicellular structures on day 2 were evaluated by the shape descriptors: area and perimeter. Area (B) and Perimeter (C) of the MCTs formed. Representative pictures of MCTs developed by ACP and HD in the presence of MC. (D Top panels showing compact structures using the ACP method, and low panels those formed with the HD method. Data are presented as mean ± SEM, (n = 5, ***p < 0.001).

Additional file 5: Figure S5. Vitality (A-C) and membrane integrity (D-F) analyses in the absence or presence of MC using the Live/Dead probes. The presence of MC did not change cell behavior. Data are presented as mean ± SEM, (n = 5). (G) Merged channels as shown singularly in A, C, D and F.

Additional file 6: Figure S6. (A) Cytotoxic core on AF spheres presented apoptotic features (green signal as measure by activation of caspase 3/7). Nuclei were counterstained with Hoechst (blue). (B-E) Impact of long-term culture in the absence of MC on cell vitality and size (ULAP condition). (B) Merges of green (vitality) and red (toxicity) channels. (C and D) Graphs depicting data of analyzed MCTs as shown in B. (C) No changes on MCTs vitality was observed between day 4 and day 7. (D) Increased cell toxicity was observed on day 7 for 92.1, OMM2.5, and MP41 cells. (E) MCTs areas were not different between day 4 and 7. Data are presented as mean ± SEM, (n = 5, ^**^p < 0.01, ^***^p < 0.001). (F-I) Impact of long-term culture in the presence of MC on cell vitality and MCTs size (ULAP condition). (F) Merges of green (vitality) and red (toxicity) channels. (G and H) Graphs depicting data of analyzed MCTs as shown in F. (I) MCTs size analyses. Increase in the total area of MCTs derived from 92.1, MEL270, and MP41 cells, which was associated with a significant increase in cell toxicity (H). In general, cell vitality is maintained during the seven days (G), the increase on vitality for OMM2.5 was associated with the increase in cell toxicity (H). Data are presented as mean ± SEM, (n = 5, ^**^p < 0.01, ^***^p < 0.001).

Additional file 7: Figure S7. Diverse morphological adaptation of UM cells on anchorage dependent culture conditions. (A) Representative phalloidin/DAPI images of MEL270, OMM2.5, and MP46 cell cultures. Arrowheads indicating clusters of irregular shapes, and different sizes. Arrows depicting few cells from OMM2.5 and MP46 that are capable to stretched acquiring a mesenchymal phenotype. (B-D) UM maintained the expression of MelanA and HMB45 markers when cultured under anchorage dependent. (B) Representative immunofluorescence microscopy images of MelanA expression on UM cells maintained under 2D culture condition. UM cells grown in 2D conditions adopted different shape phenotypes. Top panels: 92.1, MEL285, and MP46 cells adopted elongated fibroblastic-like cytoplasmic projections. Low panels MEL270, OMM2.5 and MP41 had an epithelioid phenotype. (C) Representative immunofluorescence microscopy images of HMB45 expression under 2D culture condition. (D) UM cells arrangements on AD-3D on top, immunofluorescence microscopy images displaying expression of melanocytic markers MelanA and HMB45.

## Data Availability

The datasets used and/or analysed during the current study are available from the corresponding author on reasonable request.

## Abbreviations

ACP: Agarose coated plate
AD: Anchorage-dependent
AF: Anchorage-free
BME: Basement membrane extracts
BSA: Bovine serum albumin
ddPCR: Droplet digital polymerase chain reaction
DNA: Deoxyribonucleic acid
dsDNA: Double strand DNA
ECM: Extracellular matrix
EHS: Engelbreth-Holm-Swarm
FBS: Fetal bovine serum
gBlocks: Artificial DNA fragments
gDNA: Genomic DNA
HD: Hanging drop
HDMC: Hanging drop methylcellulose
H&E: Hematoxylin and eosin
MC: Methylcellulose
MCTS: Multicellular tumor spheroids
MCTs: Multicellular tumor structure
NTC: No template control
PFA: Paraformaldehyde
PDXs: Patient derived xenografts
PBS: Phosphate buffer saline
RT: Room temperature
STR: Short tandem repeats
2D: Two-dimension
3D: Three-dimension
ULAP: Ultralow attachment plate
UM: Uveal melanoma
VEGF: Vascular endothelial factor
