# Supplementary figures and images for: Delineating three-dimensional behavior of uveal melanoma cells under anchorage independent or dependent conditions
Source: Cancer Cell Int. 2024 May 23;24:180. doi: 10.1186/s12935-024-03350-0 (PMC11118898; doi:10.1186/s12935-024-03350-0)

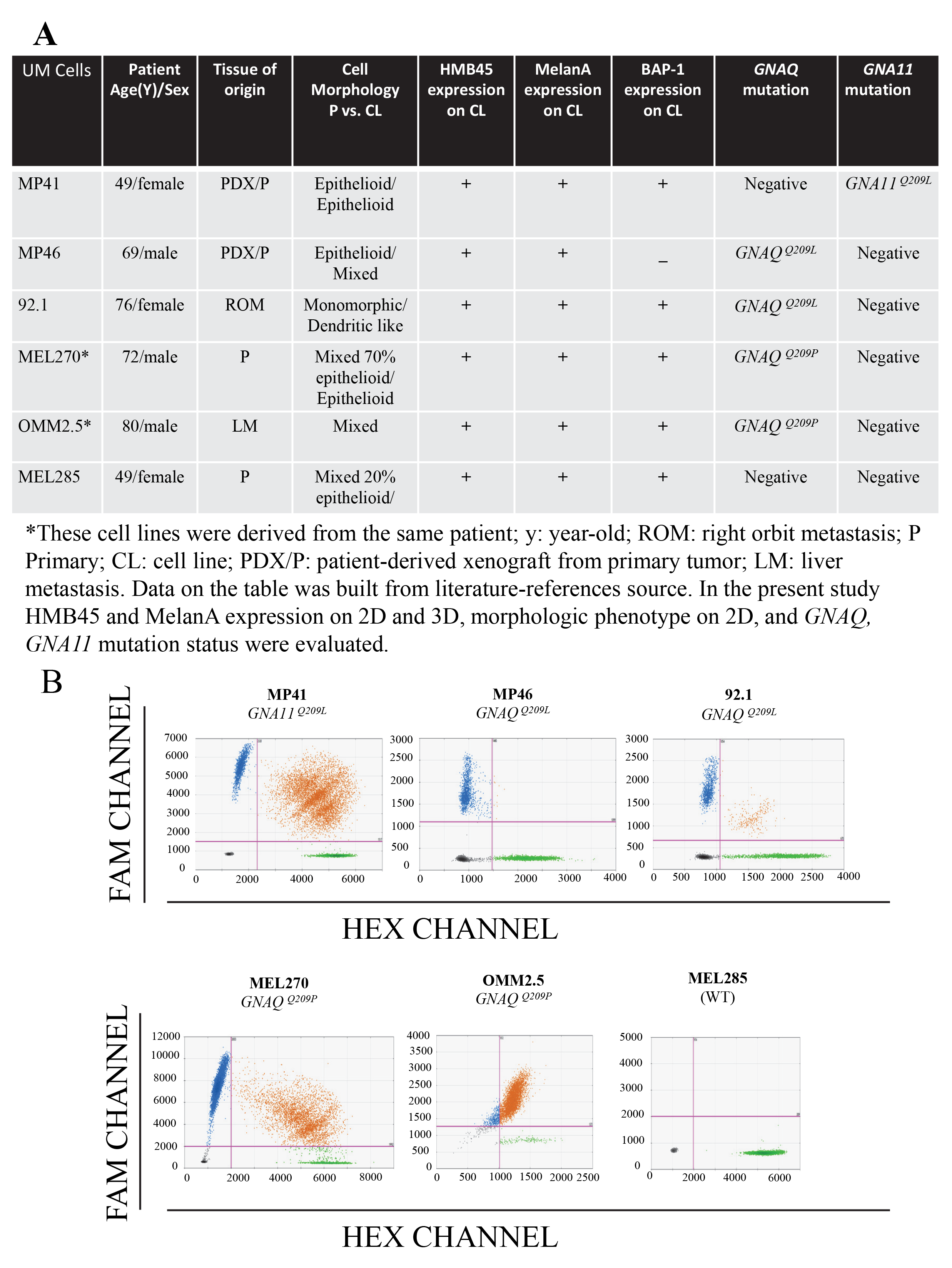

Supplement: Supplementary file 1 — Additional file 1: Figure S1. Characterization of UM cell lines used in the present study. (A) Table displaying phenotypic and genotypic characteristics of the used UM cell lines. Patients (age and sex), and tissues of origin were extracted from the literature. Expression of typical melanocyte markers as well as known GNAQ/GNA11 mutation were tested in our laboratory. (B) Representative two-dimensional plot of ddPCR showing the GNAQ/GNA11 mutation signature. Droplets positive to mutant GNAQ/GNA11 are shown in blue (FAM channel), wildtype GNAQ/GNA11 positive droplets in green (HEX channel), double positive droplets for mutant and wildtype GNAQ/11 are presented in orange, and negative droplets are represented in blank. Threshold line is presented in pink. [file 12935_2024_3350_MOESM1_ESM.tif]

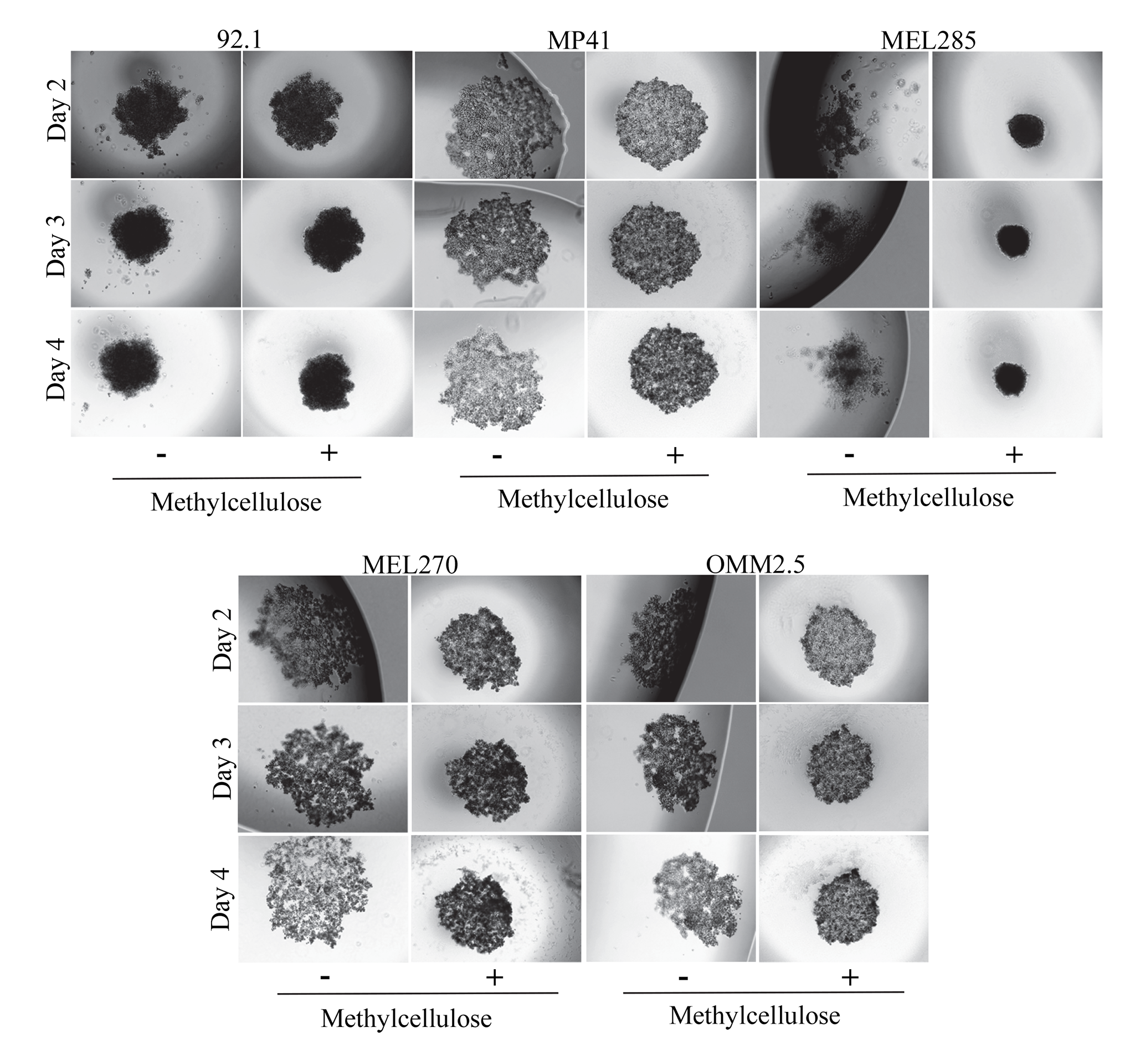

Supplement: Supplementary file 2 — Additional file 2: Figure S2. The hanging drop method without or with methylcellulose. Representative images on day 2, day 3, and day 4 of different UM cell lines showing that 5 × 10^3^ UM cells contained in 20 µl of medium and suspended under gravity as a single hanging drop developed multicellular structures (n = 3 independent cultures). Less compactness was observed when compared to the MCTs formed in the presence of methylcellulose. [file 12935_2024_3350_MOESM2_ESM.tif]

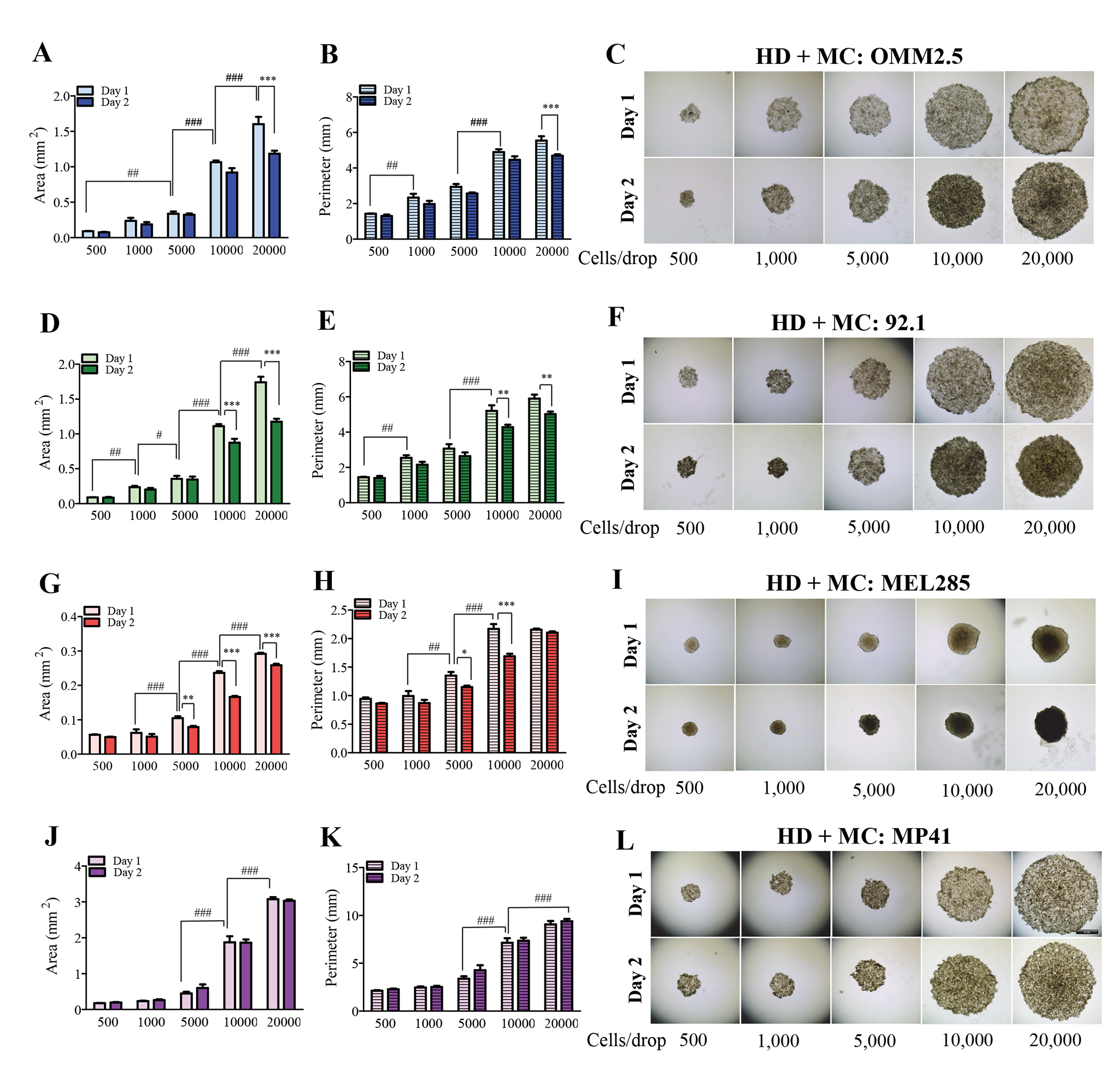

Supplement: Supplementary file 3 — Additional file 3 Figure S3. Area and perimeter to describe cell density and compactness. Contrast of multicellular structures formed after 1 or 2 days using different amounts of cells per drop (hanging drop method with methylcellulose). OMM2.5 cells (A-C), 92.1 cells (D-F), MEL285 cells (G-I), and MP41 cells (J-L) were analyzed. Cell arrangement was evaluated by MCTs Area (A, D, G and J) and Perimeter (B, E, H and K) was depicted for each cell line. Data are presented as mean ± SEM, (n = 3, ***p < 0.001, #p < 0.05, ##p < 0.01, ###p < 0.001). (C) Representative images of MCTs formed on day 1 and day 2. Note that with increased cell number seeded per drop, larger areas and perimeters were obtained denoted by #. Compactness with time, that is unique to each UM-MCTs, is demonstrated with a decrease in areas and perimeters at day 2 when compared to day 1 denoted by *. [file 12935_2024_3350_MOESM3_ESM.tif]

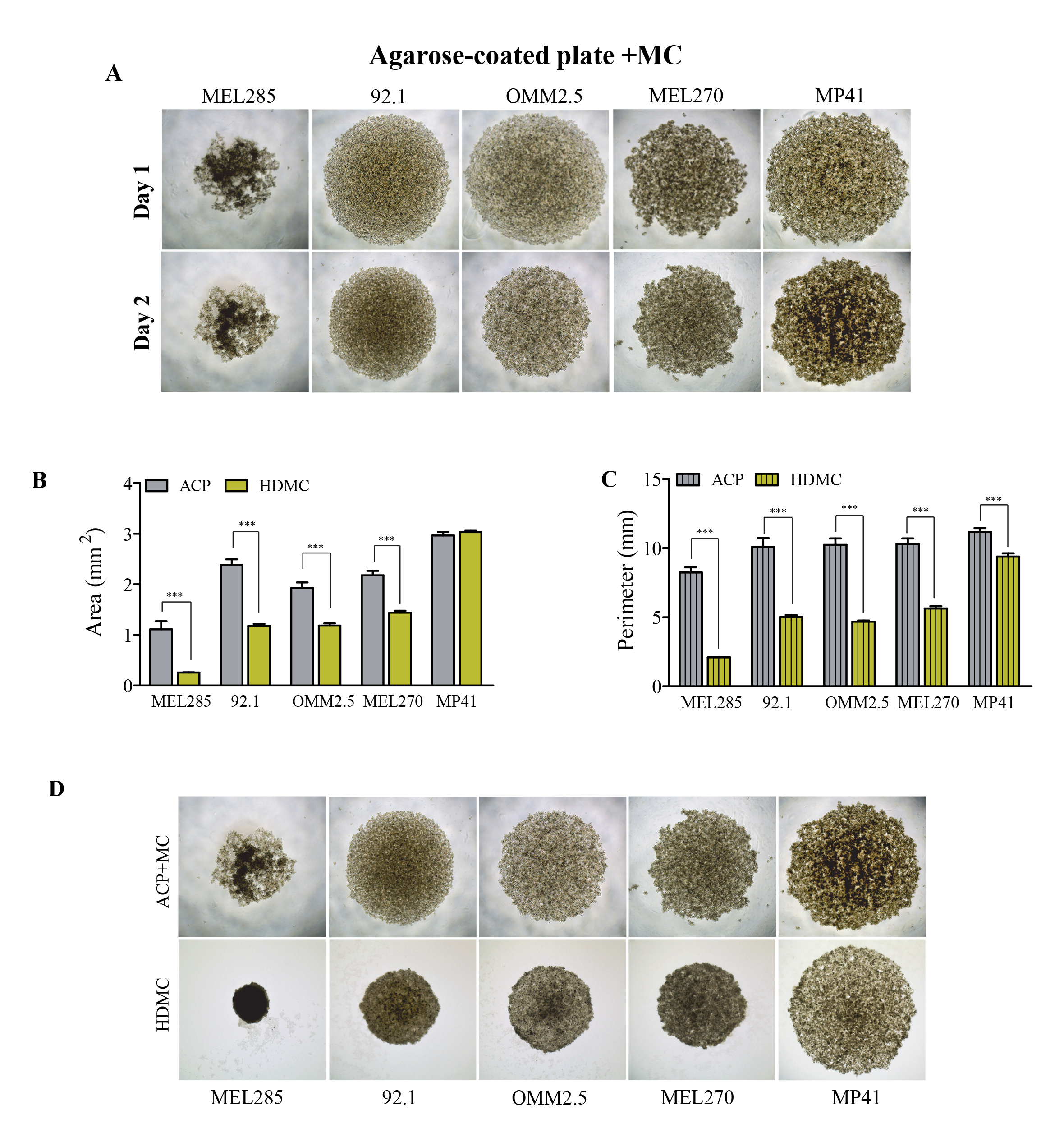

Supplement: Supplementary file 4 — Additional file 4: Figure S4. Multicellular structures formed in agarose-coated plates in the presence of methylcellulose. Area and Perimeter comparison with HDMC. (A) UM cells plated on agarose in the presence of MC developed MCTs which were more compact on day 2. (B-C) Compaction behavior of MCTs generated using the HD and ACP methods in the presence of MC. The multicellular structures on day 2 were evaluated by the shape descriptors: area and perimeter. Area (B) and Perimeter (C) of the MCTs formed. Representative pictures of MCTs developed by ACP and HD in the presence of MC. (D Top panels showing compact structures using the ACP method, and low panels those formed with the HD method. Data are presented as mean ± SEM, (n = 5, ***p < 0.001). [file 12935_2024_3350_MOESM4_ESM.tif]

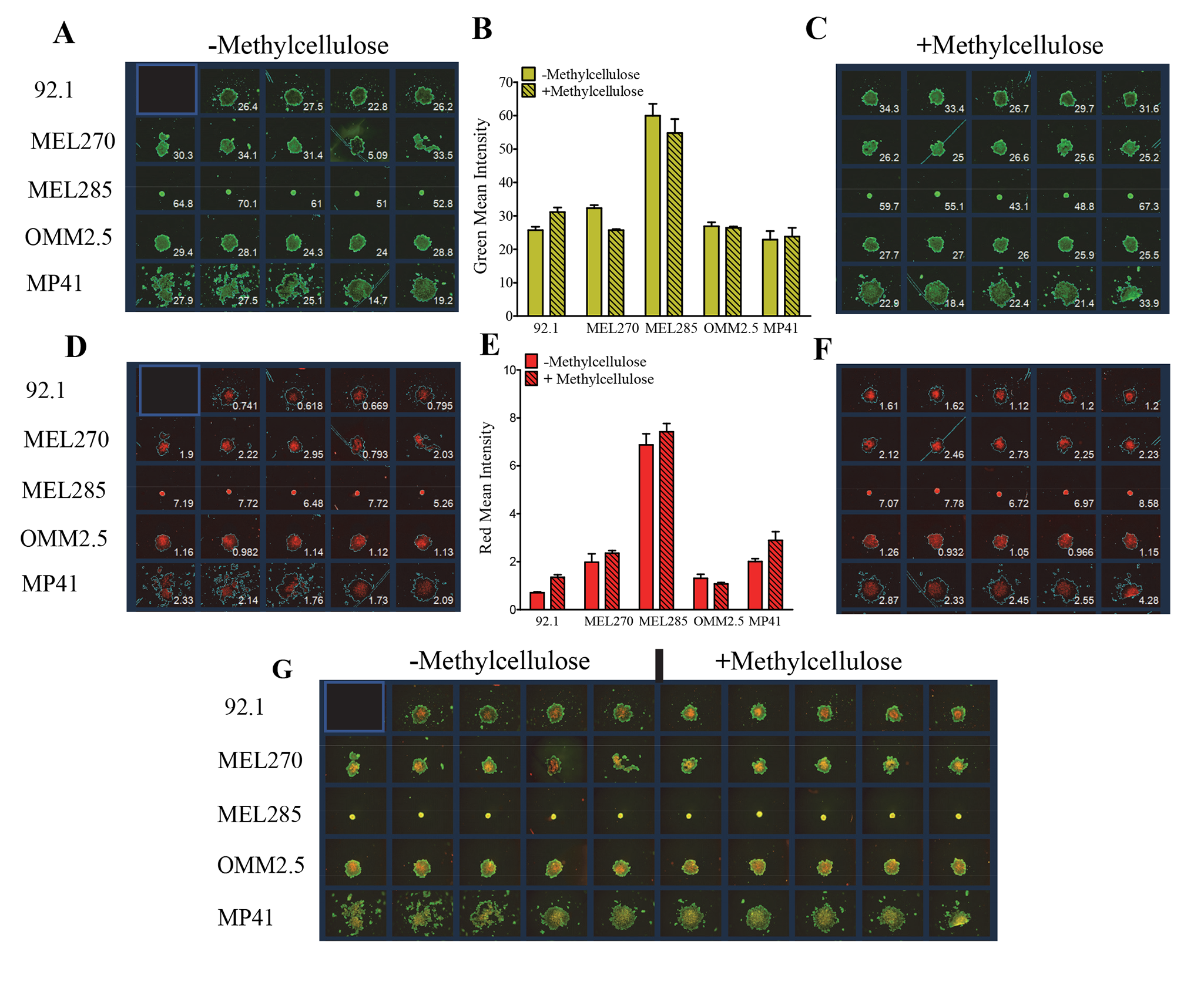

Supplement: Supplementary file 5 — Additional file 5: Figure S5. Vitality (A-C) and membrane integrity (D-F) analyses in the absence or presence of MC using the Live/Dead probes. The presence of MC did not change cell behavior. Data are presented as mean ± SEM, (n = 5). (G) Merged channels as shown singularly in A, C, D and F. [file 12935_2024_3350_MOESM5_ESM.tif]

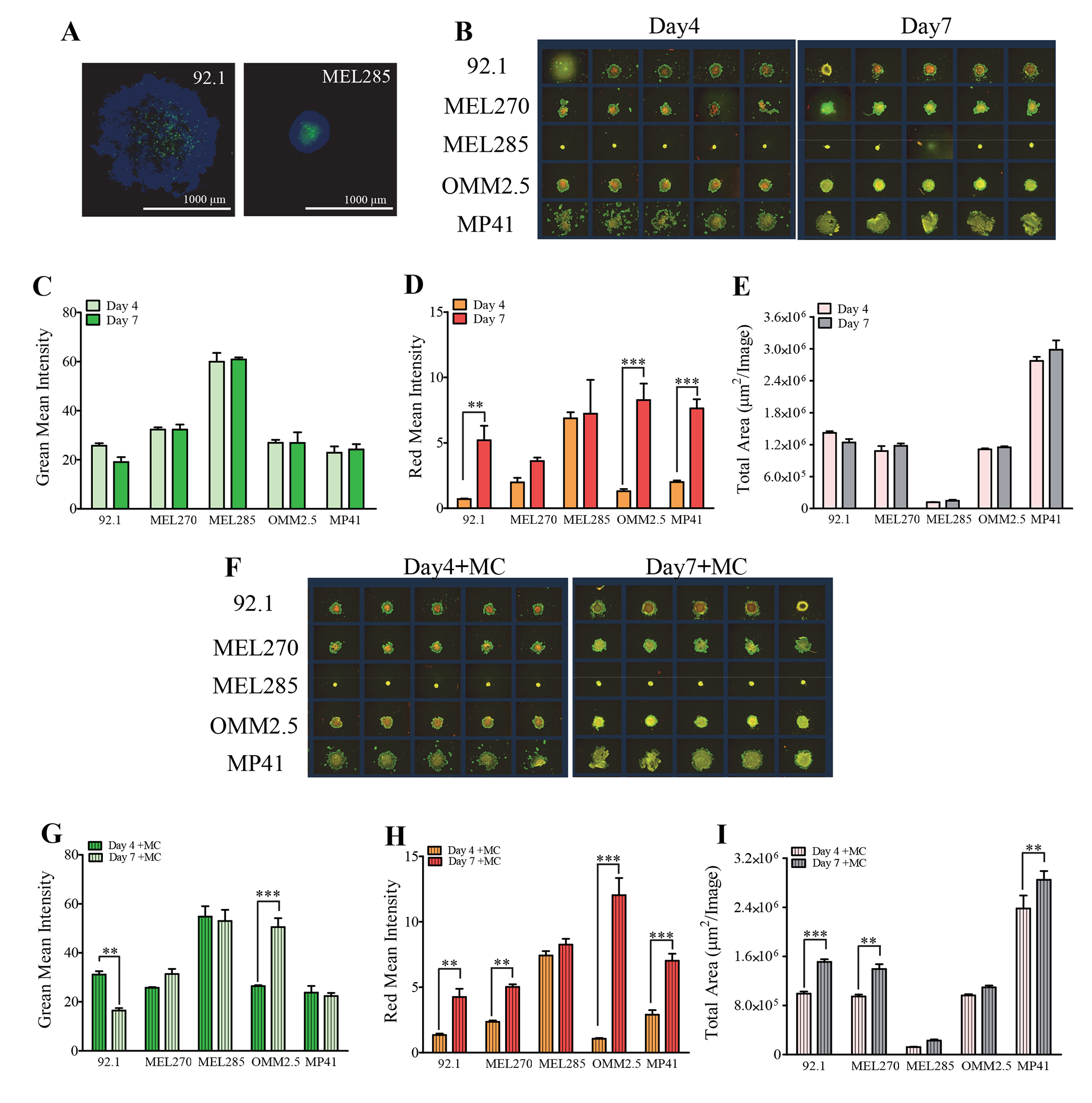

Supplement: Supplementary file 6 — Additional file 6: Figure S6. (A) Cytotoxic core on AF spheres presented apoptotic features (green signal as measure by activation of caspase 3/7). Nuclei were counterstained with Hoechst (blue). (B-E) Impact of long-term culture in the absence of MC on cell vitality and size (ULAP condition). (B) Merges of green (vitality) and red (toxicity) channels. (C and D) Graphs depicting data of analyzed MCTs as shown in B. (C) No changes on MCTs vitality was observed between day 4 and day 7. (D) Increased cell toxicity was observed on day 7 for 92.1, OMM2.5, and MP41 cells. (E) MCTs areas were not different between day 4 and 7. Data are presented as mean ± SEM, (n = 5, **p < 0.01, ***p < 0.001). (F-I) Impact of long-term culture in the presence of MC on cell vitality and MCTs size (ULAP condition). (F) Merges of green (vitality) and red (toxicity) channels. (G and H) Graphs depicting data of analyzed MCTs as shown in F. (I) MCTs size analyses. Increase in the total area of MCTs derived from 92.1, MEL270, and MP41 cells, which was associated with a significant increase in cell toxicity (H). In general, cell vitality is maintained during the seven days (G), the increase on vitality for OMM2.5 was associated with the increase in cell toxicity (H). Data are presented as mean ± SEM, (n = 5, **p < 0.01, ***p < 0.001). [file 12935_2024_3350_MOESM6_ESM.tif]

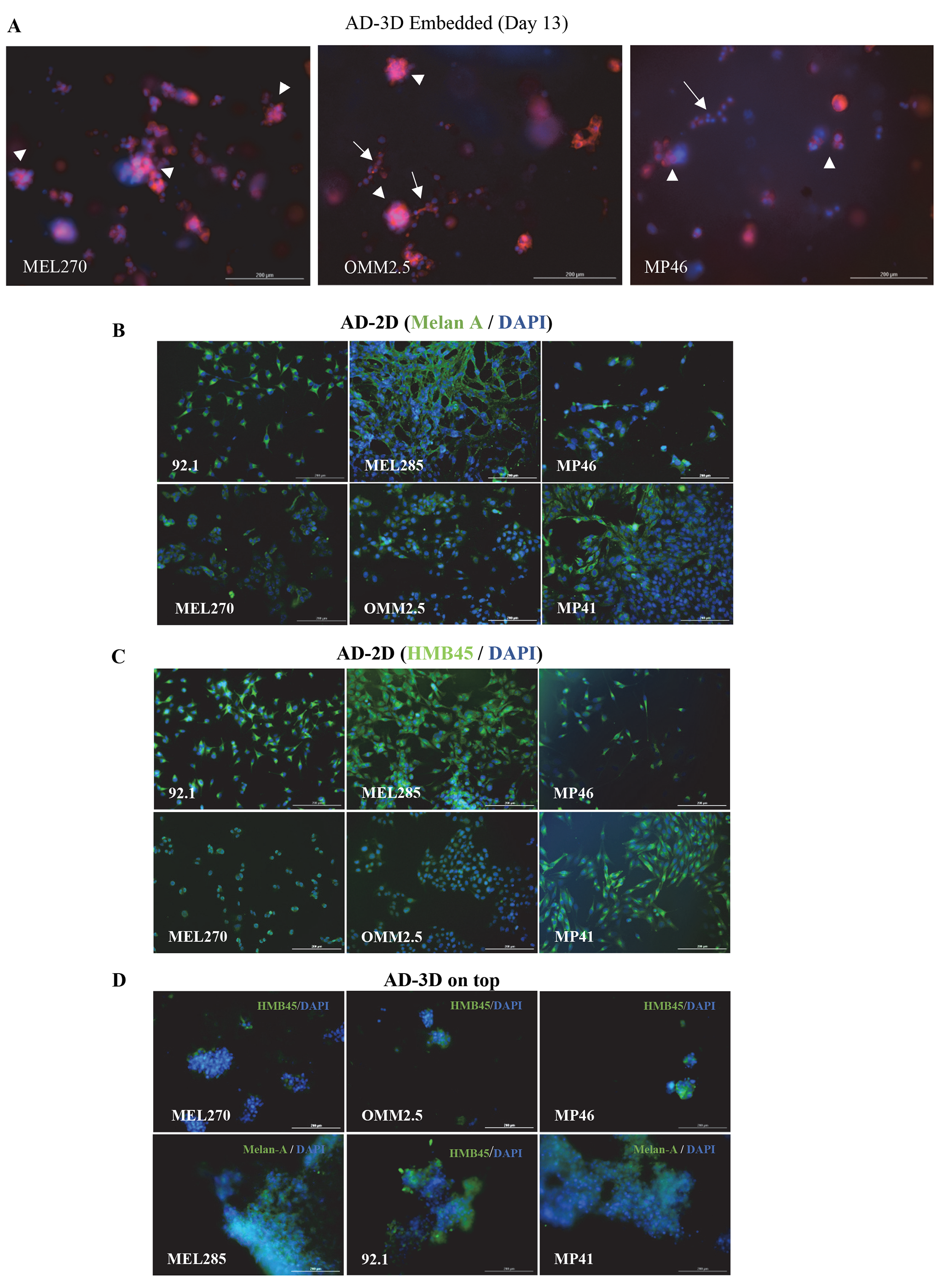

Supplement: Supplementary file 7 — Additional file 7: Figure S7. Diverse morphological adaptation of UM cells on anchorage dependent culture conditions. (A) Representative phalloidin/DAPI images of MEL270, OMM2.5, and MP46 cell cultures. Arrowheads indicating clusters of irregular shapes, and different sizes. Arrows depicting few cells from OMM2.5 and MP46 that are capable to stretched acquiring a mesenchymal phenotype. (B-D) UM maintained the expression of MelanA and HMB45 markers when cultured under anchorage dependent. (B) Representative immunofluorescence microscopy images of MelanA expression on UM cells maintained under 2D culture condition. UM cells grown in 2D conditions adopted different shape phenotypes. Top panels: 92.1, MEL285, and MP46 cells adopted elongated fibroblastic-like cytoplasmic projections. Low panels MEL270, OMM2.5 and MP41 had an epithelioid phenotype. (C) Representative immunofluorescence microscopy images of HMB45 expression under 2D culture condition. (D) UM cells arrangements on AD-3D on top, immunofluorescence microscopy images displaying expression of melanocytic markers MelanA and HMB45. [file 12935_2024_3350_MOESM7_ESM.tif]
